# Axial length of cataract eyes: a comparison of two cohorts over a span of 10 years apart

**DOI:** 10.1186/s12886-021-01859-w

**Published:** 2021-03-01

**Authors:** Yu-Ting Hsiao, Po-Chiung Fang, Pei-Chang Wu, Ming-Tse Kuo, Yi-Hao Chen, Hsi-Kung Kuo

**Affiliations:** 1 Department of Ophthalmology, Kaohsiung Chang Gung Memorial Hospital, Kaohsiung, Taiwan; 2 Chang Gung University College of Medicine, No.123, Dapi Rd., Niaosong Dist, 833 Kaohsiung, Taiwan

**Keywords:** Axial length, Myopia, Age‐related cataract, Adults

## Abstract

**Background:**

To assess the associations of axial length with age-related cataract within a span of 10 years in an Asian population in southern Taiwan.

**Methods:**

A retrospective cohort study examined 960 adults who underwent cataract surgery at the Kaohsiung Chang Gung Memorial Hospital in year 2008 and year 2018. Axial length was assessed with the ultrasound biometry and/or the Zeiss IOLMaster. Eyes with prior blunt eye trauma or had underwent vitrectomy operations were excluded. The significance of the changes in axial length between the two cohorts was determined after performing age-matched analyses. Due to utilization of ultrasound biometry and/or Zeiss IOLMaster, axial length corrections with our mean difference in measurement results, which were similar to previous studies on comparison between the two measurement tools, were carried out.

**Results:**

Axial length showed an age-related elongation in 10-year cross-sectional data, from a mean of 23.65 ± 1.80 mm in year 2008 to a mean of 24.30 ± 1.90 in year 2018 (*p* = 0.003). Patients with high myopia (axial length > 26 mm) increased significantly over the 10-year period from 8.1 to 16 % (*p* < 0.001). A birth cohort effect on axial length was evident as the axial lengths of year 2008 cohort were shorter than the 2018 cohort when they were in the same operation age group. In particular, persons born after the 1960s demonstrated a predominant increase in axial length in both cohorts.

**Conclusions:**

Our study confirms a trend in increase of axial myopia, especially high myopias, over the 10-year period. A novel finding of this study was discovering a birth cohort effect on axial length, especially in persons born after the 1960s in southern Taiwan.

## Background

Pathological myopia is one of the leading causes of visual impairment worldwide, because of complications occurring in adulthood, such as myopic macular degeneration, premature cataract, retinal detachment, and/or glaucoma. The trait results from the excessive elongation of ocular axial length [1]. It was found that the prevalence of myopia was 66.4 % higher among participants aged 12 to 54 years in the 1999–2004 National Health and Nutrition Examination Survey (NHANES) than in the 1971–1972 NHANES [2]. A rapid increase in prevalence of myopia combined with sight-threatening complications is a significant economic burden on public health, and an exponential increase in the prevalence of visual impairment with increasing age has been documented [3, 4]. In an older group of Europeans, risk of visual impairment for eyes with an axial length of 26 to less than 28 mm increased gradually for participants 60 years and older, whereas eyes with an axial length of 28 mm or greater were increasingly visually impaired at approximately 45 years and older [4].

Much attention has been focused on the prevalence of myopia in the children population, and developing strategies to prevent the development of myopia in younger generations [5–7]. However, consideration of older adults’ quality of life (QoL) has become increasingly important nowadays as there are longer life expectancies and an aging population [8]. Therefore, better understanding of the trends associated with age-related axial length changes may help reduce the burden of visual impairment in aging populations.

It is known that nuclear sclerosis of the lens may lead to a myopic shift in refraction [9]. However, whether myopia contributes to incidence of age-related cataract could not be confirmed in a meta-analysis [10]. The axial length of the eye was believed to reach adult length by the age of 13 years, therefore, it is improbable that the eye could elongate in the third and fourth decades of life [11]. The Singapore Malay Eye Study confirmed that myopia but not axial length was associated with nuclear cataract [12]. With the concept that the axial length of the eye does not increase in adults, we examined long-term changes of axial length in adults.

Despite axial length being a major variable for visual quality of the image on the retina, relatively few studies are focused on axial length. Investigations from study populations in Singapore, India, China, England and the U.S. have been performed, which were mostly population-based, cross-sectional studies, with the mean axial length of adults ranging from 22.6 mm to 23.38 mm [13–17]. Thus, the purpose of our study was to examine the 10-year change in axial length in an older southern Taiwanese population, and the relative effects of age and year of birth on axial length.

## Methods

### Study Population

A database of 1195 eyes of 1011 patients who underwent cataract surgery in a tertiary hospital in southern Taiwan, Kaohsiung Chang Gung Memorial Hospital, during January 1st to December 31st 2008, and January 1st to June 30th 2018 was studied retrospectively. Cataract surgeries were carried out by the same four experienced physicians in both years. Exclusion criteria were as follows: (1) eyes with prior vitrectomy (2) eyes with secondary cataract developed from blunt eye trauma.

**Fig. 1:**
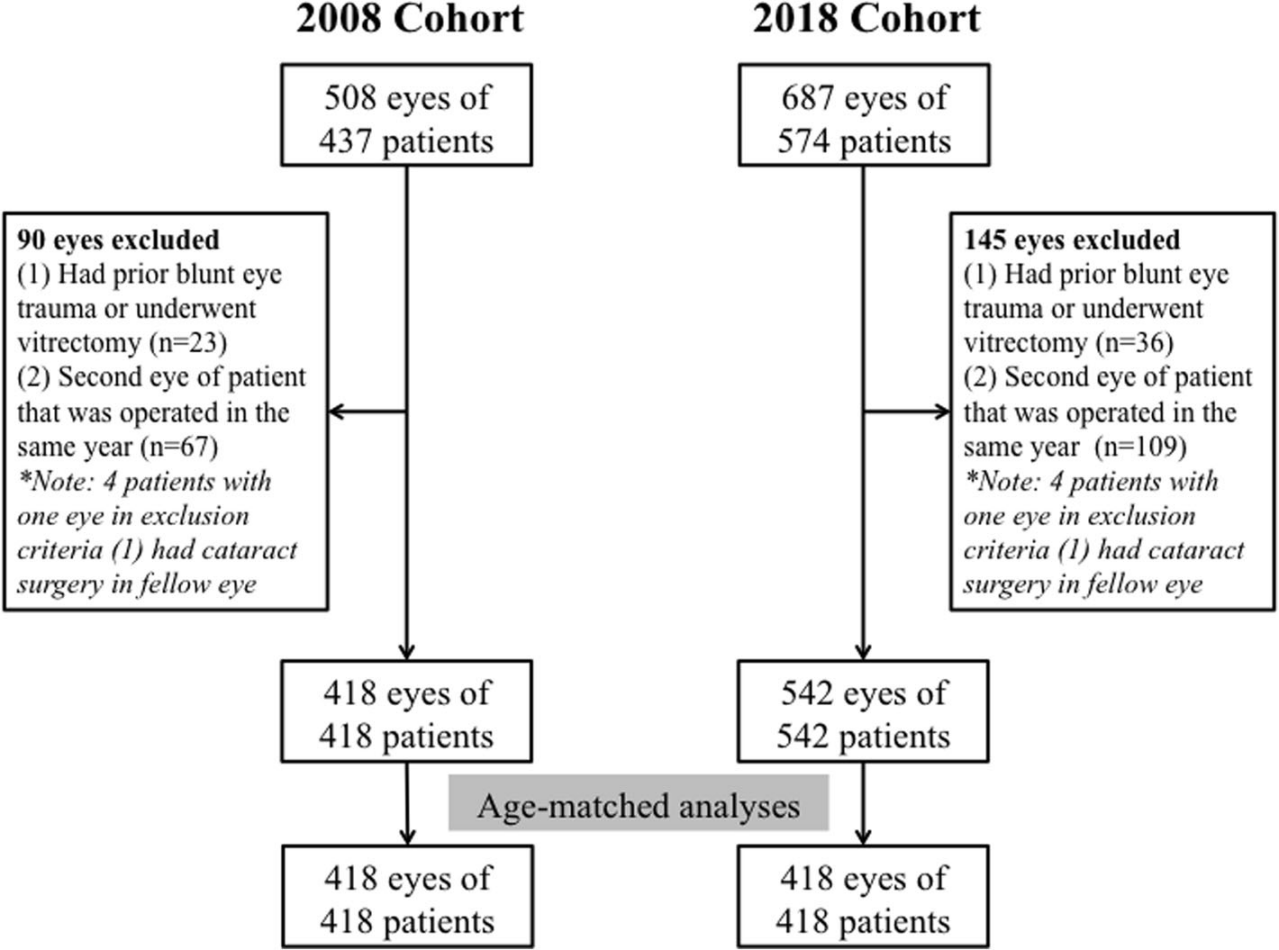
Schematic flowchart of study design

Of the 1195 eyes, 235 eyes were excluded. Fifty-nine eyes had a history of vitrectomy or blunt eye trauma. If cataract surgery was performed on both eyes in the same year by the four physicians, the first eye that underwent surgery was selected. Therefore, 176 eyes were excluded due to being the second eye that was operated in the same year. The study adhered to the Declaration of Helsinki and was approved by the Chang Gung Institutional Research Ethics Committee. Since this was a study that retrospectively reviewed medical records and there were no anonymous issues involved, the Chang Gung Institutional Review Board waived the need for consent. The study flowchart is provided in Fig. 1.

### Measurement of Axial length

All of the patients underwent a complete ophthalmologic examination before undergoing cataract surgery, including measurement of the axial length. The axial length was measured by A-scan ultrasonography (OcuScan; Alcon, Irvine, CA, USA) in cohort 2008, and by the same A-scan ultrasonography machine in 2018 and/or ocular biometry (IOLMaster 500; Carl Zeiss Meditec, Jena, Germany) in cohort 2018. The ultrasound biometry measurements were carried out using the contact method by the same group of ophthalmologists who underwent the same degree of training and had extensive experience in ultrasound biometry in each cohort relatively. During the examinations for each patient in both cohorts, the averages of the 5 most consistent results giving the lowest standard deviation (not exceeding 0.1 mm) were chosen [18–20].

### Statistical analysis

In descriptive analyses, quantitative variables were shown as mean ± standard deviation, and categorical data represented as numbers and percentages. The Gaussian distribution of the parameters was tested by the Kolmogorov-Smirnov test. Continuous variables between two groups were compared using Student’s t-test. Fisher’s exact or Pearson’s chi-squared (χ2) tests were used to compare categorical data, with odds ratios (ORs) being calculated with 95 % confidence intervals (CIs). Because the age of patients differed significantly between groups, we performed conditional logistic regression subanalyses for comparisons between age-matched Year 2008 and 2018 groups.

Correlation analyses were performed to confirm the agreement between the IOLMaster and A- scan measurements using the fellow eye of our patients. The axial lengths of cohort 2018 measured using IOLMaster were corrected after reviewing studies on comparison between axial length measured with the IOLMaster and A-scan. A P value of < 0.05 was considered to be significant. Statistical analysis was performed using SPSS 20.0 (SPSS Inc, Chicago, Illinois, USA), and NCSS 11 software (NCSS Statistical Software, Kaysville, Utah) was utilized for age-match analyses.

## Results

A total of 960 eyes (2008 cohort: 418 eyes; 2018 cohort: 542 eyes) of 960 patients were included in our analysis. Patient demographic data of both cohorts are shown in Table 1. We observed a significant increase between the 2008 cohort and 2018 cohort with respect to mean age (2008 cohort, age 65.6 ± 10.5; 2018 cohort, age 67.1 ± 10.0; *P* = 0.03), axial length (2008 cohort, 23.65 ± 1.80 mm; 2018 cohort, 24.23 ± 1.88 mm; *P* < 0.001), and number of patients with axial length > 26 mm (2008 cohort, *n* = 34; 2018 cohort, *n* = 84; *P* = 0.001). The number of patients with axial length < 22.5 mm significantly decreased (2008 cohort, *n* = 96; 2018 cohort, n = 52; *P* < 0.001) between the 2 cohorts. Moreover, the axial length of presenile cataracts with cutoff age at 55-years-old was longer in the 2018 cohort than in the 2008 cohort (2008 cohort, 25.28 ± 2.47 mm; 2018 cohort, 26.30 ± 2.57 mm; *P* = 0.039) (Table 1). No significant difference in the number of patients with presenile cataract was found.

**Table 1:** Comparison of Characteristics and Axial length of Age-Related Cataracts between Year 2008 and 2018

	Year 2008 (418 eyes)	Year 2018 (542 eyes)	Odds Ratio (95 % Confidence Interval)	*P* value
Mean age (yrs)	65.6 ± 10.5	67.1 ± 10.0		0.030
Male Gender, no. (%)	178 (42)	243 (45)	0.917 (0.709, 1.186)	0.509
Axial length (mm)	23.65 ± 1.80	24.23 ± 1.88		< 0.001
Axial length > 26 mm, no. (%)	34 (8.1)	84 (15.5)	2.071 (1.360, 3.155)	0.001
Axial length < 22.5 mm, no. (%)	96 (23)	52 (9.6)	0.356 (0.247, 0.513)	< 0.001
Age < 55, no. (%)	56 (13.4)	55 (10.1)		0.119
Age < 55, axial length (mm)	25.28 ± 2.47	26.30 ± 2.57	1.177 (1.008, 1.375)	0.039

Due to the 2018 cohort being older than the 2008 cohort, we conducted analysis using age-matched 2008 and 2018 cohorts, resulting in 418 eyes in each group respectively (Table 2). In the subanalyses between age-matched groups, mean axial length (*P* = 0.003) and number of patients with axial length > 26 mm (*P* < 0.001) were significantly higher, and the number of patients with axial length < 22.5 mm (*P* < 0.001) significantly decreased in the 2018 cohort.

**Table 2:** Comparison of Characteristics and Axial length of Age-Related Cataracts between Age-Matched Year 2008 and 2018 Groups

	Year 2008 (418 eyes)	Year 2018 (418 eyes)	Odds Ratio (95 % Confidence Interval)	*P* value
Mean age (yrs)	65.6 ± 10.5	66.1 ± 10.3		0.489
Male Gender, no. (%)	177 (42.3)	189 (45.2)		0.403
Axial length (mm)	23.65 ± 1.80	24.30 ± 1.90	1.280 (1.166, 1.406)	0.003
Axial length > 26 mm, no. (%)	34 (8.1)	69 (16.5)	2.667 (1.615, 4.403)	< 0.001
Axial length < 22.5 mm, no. (%)	96 (23)	40 (9.6)	0.309 (0.197, 0.483)	< 0.001

**Fig. 2:**
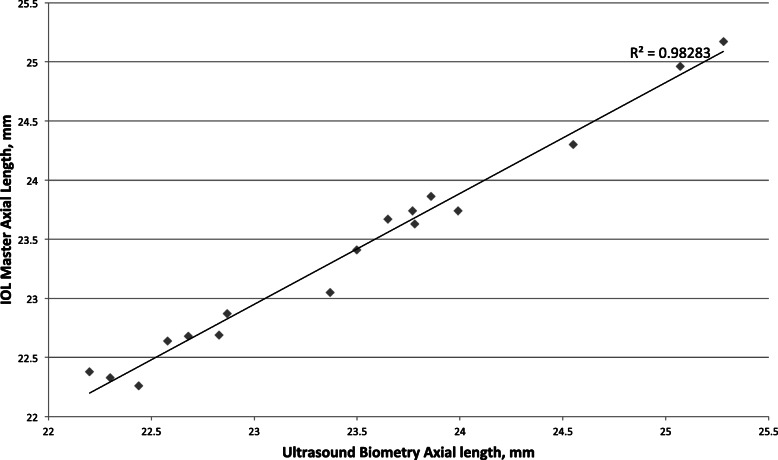
Plot of axial length measurements between the IOLMaster 500 and ultrasound biometry with a correlation coefficient of 0.98

Table 3 summarizes the axial length results of 17 patients who used both applanation A-scan ultrasound and the Zeiss IOLMaster in our study. The axial length data was found to be longer when measured with the IOLMaster. The difference was statistically significant (mean = 0.08 ± 0.13 mm, *P* = 0.023). Measurements of axial lengths using the two techniques were highly correlated (Fig. 2). Our mean difference in the measured axial length obtained with both devices correlated with the findings of previous studies reviewed in Table 3. After axial length measurements assessed with the IOLMaster were corrected (-0.08 mm), the mean axial length (*P* < 0.001), number of patients with axial length > 26 mm (*P* < 0.001), and number of patients with axial length < 22.5 mm (*P* < 0.001) remained statistically significant (Table 4).

**Table 3:** Results of Various Studies on Comparison between Mean Axial Length Measured with the IOLMaster and A-scan

	IOL Master 500 (Mean ± SD) (mm)	A-Scan (Mean ± SD) (mm)	Difference (mm)	Sample Size	*P* value
Kiss et al. 2002 [21]	23.7	23.5^a^	0.2	45	-
Németh et al. 2003 [22]	23.73 ± 2.05	23.3 ± 1.95	0.39 ± 0.36	255	< 0.001
Rose et al. 2003 [23]	23.36 ± 1.24	23.21 ± 1.30	0.15	51	0.0002
Bhatt et al. 2008 [24]	23.97	23.92	0.05	421	-
Roy et al. 2012 [25]	23.43 ± 1.06	23.23 ± 0.98	0.2	31	< 0.0001
Gaballa et al. 2017 [26]	26.18 ± 2.92	26.02 ± 2.99	0.2 ± 0.44	40	0.007
Our study	23.45 ± 0.93	23.38 ± 0.88	0.08 ± 0.13	17	0.023

^a^Immersion technique used

**Table 4:** Comparison of Characteristics and Corrected Axial Length of Age-Related Cataracts between age-matched Year 2008 and 2018 groups

	Year 2008 (418 eyes)	Year 2018 (418 eyes)	Odds Ratio (95 % Confidence Interval)	*P* value
Mean age (yrs)	65.6 ± 10.5	66.1 ± 10.3		0.489
Male Gender, no. (%)	177 (42.3)	189 (45.2)		0.403
Axial length (mm)	23.65 ± 1.80	24.23 ± 1.90	1.247 (1.139, 1.367)	< 0.001
Axial length > 26 mm, no. (%)	34 (8.1)	67 (16.0)	2.156 (1.392, 3.339)	< 0.001
Axial length < 22.5 mm, no. (%)	96 (23)	46 (11.0)	0.415 (0.283, 0.608)	< 0.001

**Fig. 3:**
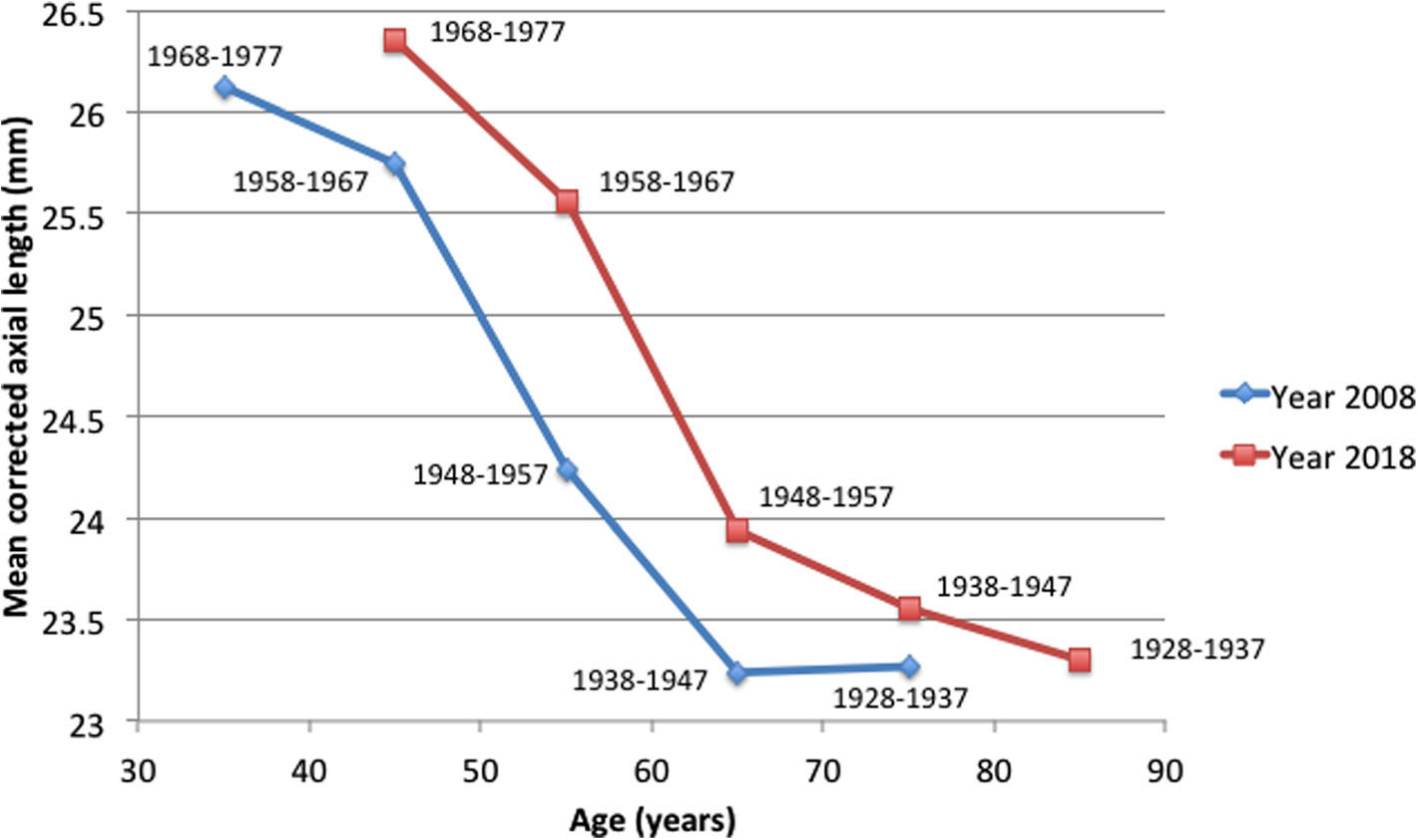
Mean corrected axial length by year of birth and corresponding age

The distribution of corrected mean axial length by age is shown in Table 5. Overall, a reduction in axial length with increasing age was observed in both cohorts, and a significant age-related trend was observed across all age groups (P < 0.001). When examining the relative effects of age and year of birth on axial length changes, a birth cohort effect on axial length was evident. The mean corrected axial length of the 2008 cohort was shorter than the 2018 cohort when they were in the same operation age group (Fig. 3). In particular, persons born after the 1960s demonstrated a predominant increase in axial length in both cohorts.

**Table 5:** Distribution of Corrected Axial Length by Baseline Age between Age-Matched Year 2008 and 2018 Groups

	Year 2008 (418 eyes)			Year 2018 (418 eyes)		
	N	Mean (95 % Confidence Interval)	P trend	N	Mean (95 % Confidence Interval)	P trend
< 55	56	25.28 (24.62–25.95)	< 0.001	49	26.12 (25.42–26.83)	< 0.001
55–64	107	23.86 (23.45–24.26)		116	24.68 (24.30-25.06)	
65–74	174	23.21 (23.02–23.40)		166	23.76 (23.55–23.98)	
75+	81	23.17 (22.96–23.38)		87	23.47 (23.24–23.70)	
All ages	418	23.65 (23.47–23.82)		418	24.23 (24.05–24.41)	

## Discussion

Various studies have shown an unprecedented rise in myopia prevalence for over half a century, especially in East Asian countries [7]. As the underlying defect of myopia is the elongated eyeball, which is also the cause of increased risk of pathological ocular complications in high myopias [27], we documented axial lengths of two cohorts over a ten-year period in an East Asian adult population. We observed an overall age-related elongation over the ten-year period, with doubling of percentage of high myopic eyes.

There has been considerable speculation about time trends in the prevalence of myopia. Although no strong evidence of time trends in myopia were found among the white population, it increased by 23 % in East Asians over the last decade [28]. In Taiwan, nationwide surveys of myopia and axial lengths in schoolchildren have been performed every 5 years since 1983, and the prevalence of myopia and high myopia has continued to increase [5]. However, there is limited data in an older, adult population. In our study, the mean age of both cohorts were in the mid-sixties, with a shift of 0.58 mm on axial length over the ten-year period. In particular, the percentage of high myopias doubled in the span of ten years from 8.1 to 16 %. Rapid economic development and stringent academic programs may have impacted the steep rise in myopia prevalence in Taiwan [5].

Population-based studies on mean ocular axial length have been performed in various regions. In a Chinese population in Singapore, Wong et al. examined 1004 phakic subjects aged 40 to 81 years of age, and a mean axial length of 23.23 ± 1.17 mm was found [14]. This was supported by the results of the Beijing Eye Study, which investigated the axial length of 3159 older individuals aged 50- to 93-year-old, and reported an average axial length of 23.25 ± 1.14 mm [13]. In 2010, Nangia et al. analyzed an adult population in rural central India, with the mean axial length at 22.6 ± 0.91 mm. The rural character of the study may be an explanation for the shorter axial length when comparing between the Indian study with previous studies [13]. A survey conducted in a general Japanese population age 40 years and older reported the trend of average axial length to be 23.4 mm in 2005 and 23.8 mm in 2017 [29]. In our study, the axial length was found to be longer than other studies at 23.65 ± 1.80 mm in the 2008 cohort and 24.23 ± 1.88 mm in 2018.

Some studies have reported age-related reductions in axial length which serves as an emmetropizing mechanism in view of the fact that the refracting power increases in the adult eye [30]. According to Gudmundsdottir et al., they observed a decrease in axial length when comparing those of 50 to 59 years of age at 23.6 ± 1.1 mm to those aged 70 years or older at 23.2 ± 1.4 mm [31]. Further verified by a study examining the 10-year change of axial length in older individuals in the Blue Mountains Eye Study, it was found that the axial length showed a decrease from 23.6 mm in 59 to 64 year olds to 23.2 mm in those aged 85 years or older [32]. In our study, we observed a significant age-related decrease in axial length across all age groups, with the difference between the youngest and oldest age group greater in the 2018 cohort than in the 2008 cohort. Furthermore, a birth cohort effect was noted when we compared the mean axial lengths of people of the same operation age group born in different periods. In the Beaver Dam Eye study, those born in more recent years were more myopic than those born in earlier years [33]. Another longitudinal study from an older population demonstrated similar birth cohort influences on spherical equivalent refraction [32].

On account of the results of this study which were obtained over a long period, only the applanation A-scan ultrasound was available in the year 2008, while the noncontact IOLMaster was more routinely used on the 2018 cohort. Some studies concluded that both contact ultrasound biometry and the IOLMaster were similar in their predictive capabilities, and while IOLMaster was an easier and faster tool to use, in eyes with significant posterior subcapsular cataracts, ultrasound biometry was still needed for accurate axial length measurement [23, 25, 34]. We analyzed axial lengths that were measured with both techniques in our data, and the mean difference in measurement results (0.08 mm) were similar to previous studies [21–26]. After axial length measurements assessed with the IOLMaster were corrected, our data in all categories still remained significant.

Strengths of our study included the standardization of the methods and the same physicians enrolled during the 10-year study period. One important limitation was the consistency in observing axial lengths across different study populations. Given the large number of participants, inclusion of all individuals who underwent cataract surgery during the study period at our institution, and our focus on a long study period of ten years, we believe our data will be generalizable to all adults seeking cataract in this population. Another was the small number of patients who had axial lengths measured with both techniques. However, as presented in Table 3, the majority of mean difference in axial length measurements with both techniques was 0.2 mm, and our difference was smaller than the majority, therefore we used the results from our data to make the axial length corrections. Third, as technological improvements and surgeon experiences may improve over time, there may be differences in the complexity of cataract surgeries between the two populations. As Kaohsiung Chang Gung Memorial Hospital is a large tertiary referral center in Southern Taiwan, patients that undergo cataract surgery at our hospital are more complex cases, both in year 2008 and 2018. We chose the same experienced ophthalmologists that performed cataract surgeries in both years to reduce the differences in surgery techniques. Lastly, further studies on axial lengths may need to be performed to correlate with the older yet general population in Taiwan.

## Conclusions

In summary, this ten-year interval cross-sectional cohort study of an older Taiwanese population provides evidence to support the hypothesis that there is a trend in increase of axial myopia, with high myopias in particular. We also observed a consistent birth cohort effect on axial length in a large tertiary hospital in southern Taiwan, especially in persons born after the1960s.

## Data Availability

The datasets used and/or analyzed during the current study are available from the corresponding author on reasonable request.

## Abbreviations

QoL: Quality of life
